# Moving an exercise referral scheme to remote delivery during the Covid-19 pandemic: an observational study examining the impact on uptake, adherence, and costs

**DOI:** 10.1186/s12889-024-19392-y

**Published:** 2024-08-27

**Authors:** Katie Newby, Neil Howlett, Adam P. Wagner, Nigel Smeeton, Olujoke Fakoya, Nigel Lloyd, Imogen Freethy, Charis Bontoft, Katherine Brown, Mary-Ann McKibben, Annie Petherick, Wendy Wills

**Affiliations:** 1 Department of Psychology, Sport and Geography, School of Life and Medical Sciences, University of Hertfordshire, Room 2H282, C.P. Snow Building College Lane Campus, Hatfield, AL10 9AB UK; 2 Norwich Medical School, University of East Anglia; National Institute for Health Research (NIHR) Applied Research Collaboration (ARC) East of England (EoE), Norwich, UK; 3 Centre for Research in Public Health and Community Care, University of Hertfordshire; National Institute for Health Research (NIHR) Applied Research Collaboration (ARC) East of England (EoE), Hatfield, UK; 4 Consultant in Public Health (Healthy Settings), Health Improvement Division, Public Health Wales, Cardiff, UK; 5 Health Improvement Division, Principal Public Health Practitioner, (Healthy Settings), Public Health Wales, Cardiff, UK

**Keywords:** Physical activity, Exercise Referral Schemes, Uptake, Adherence, Cost analysis, Observational study, Multi-level modelling, Inequalities, Virtual

## Abstract

**Background:**

Exercise Referral Schemes (ERSs) have been implemented across Western nations to stimulate an increase in adult physical activity but evidence of their effectiveness and cost-effectiveness is equivocal. Poor ERS uptake and adherence can have a negative impact on effectiveness and cost-effectiveness and, if patterned by socio-demographic factors, can also introduce or widen health inequalities. Different modes of ERS delivery have the potential to reduce costs and enhance uptake and adherence. The primary aim of this study was to examine the effect of different programmes of ERS delivery on scheme uptake and adherence. Secondary aims were to examine the effect of socio-demographic factors on scheme uptake and adherence, and the impact of delivery mode on the expected resource and corresponding costs of delivering core parts of the programme.

**Methods:**

This was an observational cohort study with cost analysis. Routine monitoring data covering a three-year period (2019–2021) from one large UK ERS (number of patients = 28,917) were analysed. During this period three different programmes of delivery were operated in succession: standard (all sessions delivered face-to-face at a designated physical location), hybrid (sessions initially delivered face-to-face and then switched to remote delivery in response to the Covid-19 pandemic), and modified (sessions delivered face-to-face, remotely, or a combination of the two, as determined on a case-by-case basis according to Covid-19 risk and personal preferences). Multi-level binary logistic and linear regression were performed to examine the effect of programme of delivery and socio-demographic characteristics on uptake and adherence. Cost data were sourced from regional-level coordinators and through NERS audits supplied by national-level NERS managers and summarised using descriptive statistics.

**Results:**

There was no effect of programme of delivery on scheme uptake. In comparison to those on the standard programme (who attended a mean of 23.1 exercise sessions) those on the modified programme had higher adherence (mean attendance of 25.7 sessions) while those on the hybrid programme had lower adherence (mean attendance of 19.4 sessions). Being older, or coming from an area of lower deprivation, increased the likelihood of uptake and adherence. Being female increased the chance of uptake but was associated with lower adherence. Patients referred to the programme from secondary care were more likely to take up the programme than those referred from primary care for prevention purposes, however their attendance at exercise sessions was lower. The estimated cost per person for face-to-face delivery of a typical 16-week cycle of the scheme was £65.42. The same cycle of the scheme delivered virtually (outside of a pandemic context) was estimated to cost £201.71 per person.

**Conclusions:**

This study contributes new evidence concerning the effect of programme of delivery on ERS uptake and adherence and strengthens existing evidence concerning the effect of socio-economic factors. The findings direct the attention of ERS providers towards specific patient sub-groups who, if inequalities are to be addressed, require additional intervention to support uptake and adherence. At a time when providers may be considering alternative programmes of delivery, these findings challenge expectations that implementing virtual delivery will necessarily lead to cost savings.

**Supplementary Information:**

The online version contains supplementary material available at 10.1186/s12889-024-19392-y.

## Background

Physical inactivity is estimated to cost the global economy 68 billion US dollars per year in healthcare costs and lost productivity [1] and is a major contributor to premature mortality [2]. Worldwide, one in four adults do not meet the recommended level of physical activity (PA) of 150 min per week of at least moderate intensity, with women reporting less activity than men [2]. An intervention which could improve these levels would provide a range of physical and mental health benefits, which would subsequently reduce a range of risk factors for serious long-term conditions. Regular PA is associated with reductions in the risk of cardiovascular disease, hypertension, stroke, cancer, and type 2 diabetes [3–6], and with improved mental health [7]. The reduction in risk for a wide range of long-term conditions is estimated to be 20–30% [6].

Some of the highest levels of inactivity are found in high-income Western countries [8]. This is true of the United Kingdom (UK) where four in ten adults do not meet the recommended levels [9]. Further, these levels differ on multiple key socio-demographic indicators that underpin existing inequalities, with women, those in older age groups, and those in the least affluent groups, all less likely to achieve the recommended amount of PA [9]. To stimulate improvements in physical activity levels and subsequent health and wellbeing, Exercise Referral Schemes (ERSs) have been implemented across Western nations. In the UK there are thought to be over 600 ERSs currently in operation [10]. Whilst variations in models of delivery exist, typical features include: 1) primary-care referral by a health professional to a service designed to increase PA; 2) delivery of a programme of exercise tailored to individual needs; and 3) initial assessment and monitoring of progress throughout the programme [11]. There has been a plethora of research examining the impacts of ERSs on changes in PA and wider outcomes such as physical and mental wellbeing. Several systematic reviews summarising this evidence report mixed conclusions [11–16]. A recent review by Campbell and colleagues [16] has good external validity due to application of the above standardised definition of ERSs for study inclusion. Pooling across five randomised controlled trials (RCTs) in which ERSs were compared with usual care, this review found only a small effect in favour of the intervention, with 12% (95% confidence interval (CI): 4% to 20%) more ERS participants achieving 90–150 min of at least moderate-intensity PA per week at 6–12 months follow-up than usual care participants. This review also examined the effect of ERSs on several outcomes in addition to PA. Whilst there was some evidence of a positive effect on psychological wellbeing, evidence in favour of improvements in blood pressure, obesity indices, respiratory function, and health-related quality of life was equivocal.

Evidence with respect to the effectiveness of ERSs and whom they benefit informs decision-making around ERS implementation and whether they should be targeted at specific sub-groups of the population. Equally important is evidence concerning cost-effectiveness. Anokye and colleagues conducted a detailed estimate of the cost-effectiveness of ERSs in the UK from the perspective of the National Health Service (NHS) which is informative in this respect [17]. They reported a favourable, albeit modest, cost-effectiveness ratio of £20,876 per quality-adjusted life-year (QALY; cost year 2010) from ERSs compared to usual care. This estimate of cost-effectiveness (incremental cost-effectiveness ratio) for inactive adults without a documented health condition is just above the threshold of £20K per QALY often taken to indicate cost-effective use of NHS resources; in the range £20-30K per QALY, other, broader, considerations may be noted before an intervention is recommended for funding[18]. Further, subgroup analysis indicated that cost-effectiveness could be improved if ERSs were targeted at individuals living with conditions known to benefit from increased PA—namely obesity, hypertension, or depression [19]. For the last group, the figure was calculated as low as £8,414 per QALY. A degree of caution is however required in drawing conclusions concerning the cost-effectiveness of ERSs. A systematic review of reviews examining the cost-effectiveness of PA interventions [20], concluded that the evidence for ERSs was inconclusive. The authors warned that cost-effectiveness will crucially depend on how costly these schemes are to deliver and advised that delivery cost is carefully considered prior to implementation.

As set out above, there is some tentative evidence that ERSs are both effective and cost-effective. Effectiveness and cost-effectiveness are both compromised however if levels of uptake and/or adherence are poor. Clearly, if people referred to ERSs do not take up the programme in the first place, then the beneficial effects of increased PA will not occur. Further, if adherence is low then outcomes will be negatively impacted [21]. With respect to cost-effectiveness, given that a large proportion of the costs of ERSs are incurred early in the scheme, low uptake or drop-out may increase costs and reduce effectiveness, negatively impacting on overall cost-effectiveness. Given this, it is important to understand what patient and programme-level factors might influence uptake and adherence. This evidence enables scheme organisers to assess whether limited resources are being appropriately and equitably used, and to put strategies in place to ameliorate these where required.

Within the literature evaluating ERSs, uptake is variously defined as attendance at a first consultation or a first exercise session, and adherence as the completion of a set number of exercise sessions or attendance at a final consultation [10]. Whilst rates of uptake and adherence are unknown for real-world ERSs currently being delivered in the UK, evidence from observational studies provides an indication. In one systematic review, pooled ERS uptake and adherence rates from observational studies were reported as 66% (95% CI: 57% to 75%) and 49% (95% CI: 40% to 59%) respectively [10]. A growing body of evidence points to factors which may influence these rates. A systematic review by Pavey and colleagues [22] summarised the predictors of ERS uptake and adherence, and subsequent work across one additional trial [21] and three observational studies [23–25] further adds to this picture. In terms of sex, there is reasonably consistent evidence that women are more likely to take up an ERS than men with five studies reporting this [21, 25–28], although two studies [29, 30] found no association. Data on sex and adherence are more limited, with one study reporting that men are more likely to adhere than women [31] but another that women are more likely to adhere than men [32]. A clear pattern is emerging for age, with increasing age reported as a predictor of increased uptake in six studies [21, 25, 26, 28, 33, 34] and of adherence in four studies [21, 28, 32, 35]. Three studies have however found no association between age and uptake [27, 36, 37]. Evidence concerning deprivation is also reasonably consistent, with four studies reporting that those living in the most deprived areas were less likely to take up ERSs [21, 25, 28, 33] and two studies reporting deprivation as a predictor of non-adherence [21, 28]. Two studies have however found no association between deprivation and adherence [26, 29].

While evidence concerning the influence of individual-level factors on ERS uptake and engagement is growing, broader factors concerning the way in which a programme is structured or delivered have received little attention [38]. ERS programmes typically involve the delivery of face-to-face exercise sessions, led by a specialist instructor, in a leisure or gym setting. Research has however identified barriers to engaging with ERSs in this format such as travelling distance, perceived safety of the location, difficulties reaching the location using public transport, cost of travel, and the timing of sessions clashing with work or childcare commitments [39]. ERS delivery using remote methods that enable attendees to exercise in their own home could help to address some of these barriers and in particular support engagement among typically underserved groups. These methods include following pre-written exercise plans, accessing pre-recorded demonstrations, and using video conferencing interfaces to participate in live classes. Whilst these remote modes of delivery may facilitate users to overcome the barriers that have been identified [39], they may also disrupt aspects of ERSs that are associated with attendance such as support from peers and providers, enjoyment, perceptions of safety, and exercising in the company of others [39]. Virtual modes of delivery also have potential to widen existing inequalities through digital exclusion, which could skew uptake and outcomes in favour of those who are least deprived [40, 41].

The National Exercise Referral Scheme (NERS) is an ERS that has been running across Wales since 2007. Like the rest of the UK, PA levels in Wales are low, with just over half of the adult population reporting that they meet the guideline levels [42]. Also mirroring UK patterns, women, those aged over 65, and people living in areas within the bottom 40% of deprivation scores, do not reach the 50% participation rate for the recommended amount of PA [42]. The NERS model, funded through the Welsh Government and managed by Public Health Wales (PHW; an NHS organisation given the remit of protecting and improving population health and wellbeing and reducing health inequalities across Wales), delivers standardised exercise referral across all 22 local authority areas. To be eligible for NERS, patients must be aged 16 years or over, sedentary, and at risk of or with an existing chronic condition. NERS aims to support service users to change physical activity behaviour and make improvements in outcomes such as their physical and mental wellbeing and motivation for self-care. In March 2020, as a result of the Covid-19 pandemic, NERS adapted to remote delivery so that patients could continue to access the scheme. This change afforded the opportunity to examine the impact of implementing different programmes of delivery on scheme uptake and engagement. The number of referrals to NERS continues to grow, leading to concerns that demand is beginning to outstrip capacity. Further, there is evidence of inequalities in the uptake of NERS which mirror those seen across other ERSs [25]. Offering all or part of the scheme in a remote format has the potential to increase capacity whilst also increasing accessibility and the type of people who can be supported. Doing so however needs to be informed by an assessment of any potential negative effects on uptake and adherence, and any negative impacts on health inequalities.

The primary aim of the present study was to examine:

- the effect of different programmes of delivery on scheme uptake and adherence.

Secondary aims were to examine:

- the effect of socio-demographic factors on scheme uptake and adherence
- the impact of delivery mode on expected resource and corresponding costs of delivering core parts of the scheme

## Methods

### Design

An observational cohort study (analysis of routine monitoring data collected by NERS) with cost analysis. The study is registered on Research Registry (researchregistry7842).

### Context, setting and participants

NERS is an example of an ERS as defined by Pavey and colleagues [10]. General Practitioners (GPs), physiotherapists, practice nurses, and other NHS registered health professionals can refer patients to the scheme. The referring professional provisionally assigns the patient to one of eleven pathways of care [see Supplementary Material 1]. Seven of these pathways are grouped as ‘level 4’ and are primarily used to support the ongoing rehabilitation of patients leaving secondary care. Individuals are usually assigned to the remaining pathways via primary care, with the non-specific ‘generic’ pathway typically used for primary prevention of chronic conditions. In each area, the scheme is delivered by a team of NERS Exercise Referral Professionals (ERPs) who operate across a variety of settings, such as council-owned leisure centres, private gyms, and community centres. At scheme commencement, there is an initial consultation where baseline measurements of physical activity and health are taken, and goals are agreed between the ERP and patient. This is followed by a check-in at around 4–8 weeks, and then two further consultations at 16 weeks and 12 months when measurements are repeated. All measurements taken are recorded on a national NERS database and used to monitor key performance indicators. The scheme itself consists of 16 weeks of supervised exercise sessions after which patients are sign-posted to alternative exercise opportunities available locally or offered a further round of the scheme (those on level 4 pathways only). Patients are asked to commit to two sessions per week and to pay a subsidised fee of £2 per session.

Prior to March 2020, NERS was delivered to patients face-to-face, via gym and group exercise sessions (throughout this paper, we refer to this form of delivery as the ‘standard’ programme). In response to the Covid-19 pandemic, face-to-face delivery switched to remote support whereby patients had the choice of attending virtual exercise classes (pre-recorded or live) and/or following a home exercise plan. The decision was made by PHW not to charge patients for remote sessions. Patients who had been receiving face-to-face exercise sessions for a minimum of four weeks were asked if they would like to switch to remote delivery or to postpone their place on NERS until face-to-face delivery returned. Those with fewer than four weeks of face-to-face sessions, or who did not want remote support, were put on to a waiting list. Throughout this paper, patients who decided to switch from face-to-face to remote support are referred to as on the ‘hybrid’ programme. From March 2021, the hybrid programme was replaced with the ‘modified’ programme, whereby delivery was determined on a patient-by-patient basis; a programme could consist of face-to-face sessions only, virtual sessions only, or a mixture of both virtual and online sessions. This arrangement was agreed between the ERP and the patient at the first consultation based on the level of Covid-19 risk [see Supplementary Material 2 for decision making matrix] and personal preference. Virtual sessions continued to incur no charge, but delivery was adapted such that classes were restricted to eight patients and had to have two ERPs present for safety purposes. See Table 1 below which summarises the characteristics of each type of programme delivery.

**Table 1:** Characteristics of each type of programme delivery

Programme contact points	Programme of delivery		
	Standard	Hybrid	Modified
Referral	Service user offered the standard programme	Service user offered the standard programme	Service user offered the modified programme
First consultation	Held face-to-face (service user received induction onto standard programme)	Held face-to-face (service user received induction onto standard programme)^a^	Held face-to-face or virtually (in line with decision making matrix presented in Supplementary Material 2 and patient preferences)
First exercise session	Held face-to-face	Held face-to-face^a^	
Remaining sessions		Changed from face-to-face to remote delivery at some point between 4–16 weeks (after which virtual or home-programme received)^b^	
Last consultation		Held remotely (either virtual or telephone)	

^a^Patients in the hybrid group were enrolled onto the programme pre-Covid and thus received their first consultation and first exercise session face-to-face, and at these points were expected to go on to receive a fully face-to-face version of the programme

^b^Only patients who had received a minimum of four weeks of face-to-face sessions were allowed to be supported remotely (other patients were put on a waiting list to continue the scheme when face-to-face lessons returned). The point at which the change from face-to-face to remote delivery occurred varied by patient, according to the point at which they were in their 16-week schedule of lessons when the scheme moved to remote delivery (in March 2020)

In March 2021, an evaluation of the impact of programme delivery on NERS uptake, adherence, and cost commenced. This was led by PHIRST (Public Health Intervention Responsive Studies Team) Connect, a research team funded by the National Institute for Health and Care Research (NIHR), consisting of academics, public contributors, and an independent study advisory board. To examine the impact on uptake and adherence, a sample of data from the NERS database was extracted and analysed. This covered a period over which all three programmes of delivery (standard, hybrid, and modified) were provided in succession.

### Study data

Data concerning patients referred between 1^st^ January 2019 and 9^th^ December 2021 were downloaded from the NERS database by the data custodian (the Welsh Local Government Association; providing oversight of NERS operational management at that time). At download, patient names were removed by the data custodian and replaced with a unique identifier. Next, each patient was assigned to a Welsh Index of Multiple Deprivation (WIMD) quintile [43] using postcode data which were subsequently deleted. WIMD is the Welsh Government’s official measure of deprivation. It draws on several indicators to provide an index of the relative deprivation in different areas in Wales. The WIMD score can be split into quintiles, with quintile 1 being the most deprived and quintile 5 being the least. After assigning WIMD quintiles, the anonymised data were transferred to the research team who prepared it for analysis. This included identifying and removing data corresponding to repeat referrals (*n* = 612), duplicate entries (*n* = 380), and referrals outside of the specified date period (*n* = 719) or with no referral date (*n* = 5) [see Supplementary Material 3 for data screening flowchart].

Within the present study, uptake was measured in two ways: attendance at the first consultation, and attendance at the first exercise session. Adherence was also measured in two ways: as the mean number of exercise sessions attended per week, and as attendance at the last consultation at 16 weeks. Programme of delivery (standard, hybrid, or modified) was determined according to the status recorded for each patient in the NERS database (completed scheme, withdrew from scheme, did not take up scheme following referral) and the timing of referral/first consultation and programme endpoint in relation to suspension of the service. Further information on how data recorded in the NERS database was used to assign individuals to each programme of delivery is available on request from the corresponding author.

For the cost analysis, the total cost of the scheme per person, when delivered either face-to-face or virtually, under the different programme types in operation (where relevant), was calculated. To provide a more complete picture, the cost of virtual delivery under a hypothetical post-pandemic version of the scheme, in which additional cleaning is not required and virtual exercise sessions incur a £2 charge, was also calculated. Data required to perform the cost analysis were obtained from multiple sources. For each mode of delivery and in accordance with the requirements of each programme type, four NERS coordinators provided information on the duration of key programme activities and the type of staff who delivered these. National-level NERS managers additionally supplied data collected internally for reporting purposes. This included in-person exercise session attendance and viewing figures for the pre-recorded exercise sessions, used to derive average class sizes for each mode of delivery, and information on annual salary costs from which hourly costs of employment were derived. See Supplementary Material 4 for further detail.

### Statistical analysis

Analyses to examine programme uptake and adherence were conducted in SPSS V27.0 (IBM Corp. Released 2020. IBM SPSS Statistics for Windows, Version 27.0. Armonk, NY: IBM Corp). First, summary statistics were calculated to describe patient characteristics. Where the dependent variable was dichotomous, predictors were examined using multi-level binary logistic regression. Where the dependent variable was continuous, multi-level linear regression was used instead, with dummy variables created for independent categorical variables. A multi-level modelling approach [44] was employed as the data were hierarchical in nature [45], with patients nested within local authorities. On each occasion, a random intercepts model was used to adjust for the impact of expected correlation between individuals within the same local authority. This ensured that any dependencies within observations on the dependent variables were accounted for. Individual-level predictors included programme of delivery, age, sex, WIMD quintile, and programme pathway.

Where the dependent variable was uptake (either measured as attendance at first consultation or at first exercise session), programme of delivery with only two levels was entered into the model: 1) standard programme, and 2) modified programme. The hybrid programme of delivery was not included because all patients in this group had, by virtue of their categorisation, already taken up the scheme. As previously stated, only patients who had attended the first consultation and received a minimum of four exercises sessions pre-pandemic could choose to move to remote delivery when the pandemic restrictions came into force. All remaining patients who chose not to take up virtual delivery (not included in the analysis) were put onto a waiting list for when face-to-face delivery resumed. See Table 1 for characteristics of each type of programme delivery.

Costs to the service of delivering the scheme under the different modes of delivery (face-to-face and virtual) within the context of each programme type were compared. Activity durations were multiplied by hourly rates to calculate costs of delivery, and then divided by class sizes to determine delivery cost per service user for each modality. The costing perspective was that of NERS and costs are reported in pound sterling based on 2020/2021 values. See Supplementary Material 4 for further detail.

## Results

### Descriptive statistics

Between January 2019 and December 2021, 37,960 patients were referred to NERS (see Supplementary Material 5 for characteristics of all patients referred). Prior to analysis, the following patients were removed from the dataset; those under 16 years of age (*n* = 25), recorded as ‘inappropriate referral’ (*n* = 453), or recorded as ‘on waiting list’ (*n* = 3,201). Also removed, were patients recorded as ‘on programme’ (*n* = 3,063) or at ‘4–8 weeks consultation’ (*n* = 1,932), and those without a date for their programme endpoint (*n* = 369) [see Supplementary Material 3 for data screening flowchart]. These patients were removed as without this information the programme of delivery they had received could not be reliably assigned. The resulting dataset contained 28,917 patients. Table 2 below displays the characteristics of the sample used in the analysis.

**Table 2:** Characteristics of sample (*n* = 28,917)

**Characteristic**		
	**Mean**	**Standard deviation**
**Age (years)**	55.8	17.2
	**Minimum**	**Maximum**
**Age (years)**	16	100
	**Frequency**	**%**
**Mode of delivery**		
Standard	22,750	78.7
Hybrid	4,168	14.4
Modified	1,999	6.9
**Sex**^a^		
Female	18,260	63.1
Male	10,656	36.9
Missing	1	
**WIMD quintile**		
1 (most deprived)	5,512	19.6
2	5,818	20.7
3	5,997	21.3
4	5,956	21.1
5 (least deprived)	4,884	17.3
**Local health board**		
Aneurin Bevan	4,973	17.2
Betsi Cadwallader	8,787	30.4
Cardiff and the Vale	4,023	13.9
Cwm Taf	4,151	14.4
Hywel Dda	3,385	11.7
Powys	1,206	4.2
Swansea Bay	2,392	8.3
**Pathway**		
Back care	1,069	3.7
Generic	16,361	56.6
Level 4^b^	4,840	16.7
Mental health	2,189	7.6
Weight management	4,458	15.4
**Referrer type**		
GP	11,368	39.3
Physiotherapist	9,417	32.6
Practice nurse	3,510	12.1
Other	4,621	16.0
Missing	1	

^a’^Sex’ rather than ‘gender’ recorded in NERS database

^b^Level 4 includes those on the following pathways: cancer, cardiac, falls prevention, lifestyle, pregnancy, pulmonary and stroke (see Supplementary Material 1)

### Inferential statistics

The following sections present the results of the multi-level analysis. Table 3 below provides a summary of these findings.

**Table 3:**
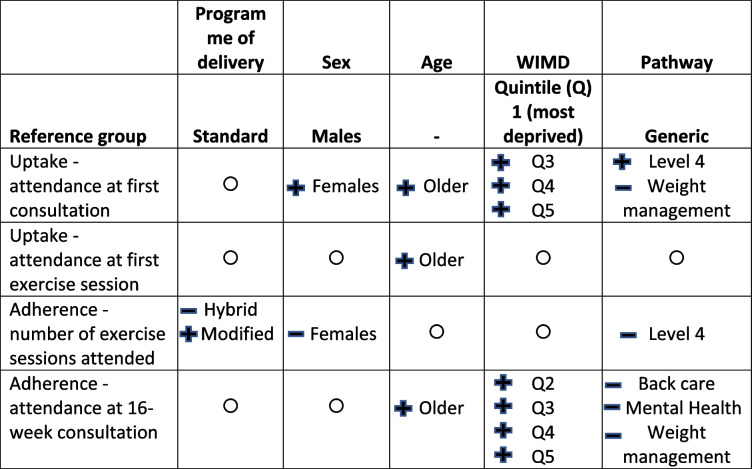
Summary of findings: tests of association between independent (programme of delivery and socio-demographic factors) and dependent (programme uptake and adherence) variables

No significant findings

Outcome more favourable for this group in comparison to reference group

Outcome less favourable for this group in comparison to reference group

#### Uptake: attendance at first consultation

As specified in the Methods section, only patients referred on to the standard and modified programmes were included in this analysis. The total number of patients referred on to either of these two programmes (*n* = 24,749) and who attended the first consultation was 10,236 (41.4%). The breakdown by programme of delivery was as follows: standard programme 9,347 (41.1%), and modified programme 889 (44.5%).

Findings from the multi-level binary logistic regression are displayed in Supplementary Material 6 (Supplementary Table 1). Programme of delivery had no effect on attendance, with those referred on to the standard and modified programmes equally likely to attend the first consultation. Attendance at the first consultation was however more likely for females than males (OR = 1.10, 95% CI: 1.01, 1.20) and increased with age (regression coefficient (b) = 0.019, 95% CI: 0.016, 0.021). There was also an upwards trend in uptake across the WIMD quintiles, with the odds of attendance increasing between WIMD quintiles three to five. Relative to the generic pathway, the odds of uptake were higher for those on a level 4 pathway (OR = 1.24, 95% CI: 1.04, 1.48) but lower for those on the weight management pathway (OR = 0.83, 95% CI: 0.69, 0.99). See Supplementary Material 7 for graphs displaying the probability (logit) of attending the first consultation by sex, pathway, and WIMD quintile.

#### Uptake: attendance at first exercise session

Only participants on either the standard or the modified programme, and who attended the first consultation, were included in analysis examining attendance at the first exercise session (*n* = 10,236). Of these, 9,096 (88.9%) attended the first exercise session. The breakdown by programme of delivery was as follows: standard programme 8,284 (88.6%), and modified programme 812 (91.3%).

Findings from the multi-level binary logistic regression examining predictors of uptake 2 are displayed in Supplementary Material 6 (Supplementary Table 2). As with the first measure of uptake, there was no effect of programme of delivery on attendance, indicating that those referred onto the standard and modified programmes were equally likely to attend the first exercise session. Also, in line with the first measure of uptake, there was a positive association between age and attendance (b = 0.012, 95% CI: 0.006, 0.017), but no effect was observed for WIMD quintile, sex or pathway.

#### Adherence: number of exercise sessions attended

Only patients who completed NERS were selected for analysis (*n* = 8,313). Of these, patients recorded as attending zero sessions (*n* = 1,845) were excluded, leaving 6,468 for analysis. The mean (M) number of sessions attended was 22.3 (standard deviation (SD) = 11.0) out of a recommended 32 (two per week for 16 weeks). Supplementary Material 8 displays the distribution of sessions attended. Nearly half of the patients in the sample (*n* = 2,800; 47.4%) attended at least 75% of sessions. This threshold level of adherence was reached by 50.6% (*n* = 1,977) of patients on the standard programme, 36.7% (*n* = 597) of patients on the hybrid programme, and 59.9% (*n* = 229) of patients on the modified programme.

Supplementary Material 6 (Supplementary Table 3) presents the results of the multi-level linear regression analysis. Programme of delivery was a significant predictor of engagement. In comparison to those on the standard programme (mean number of sessions attended = 23.1, SD = 10.6), those on the hybrid programme attended fewer exercise sessions (mean number of sessions attended = 19.4, SD = 11.4; b = -3.81, 95% CI: -4.45, -3.16), and those on the modified programme attended more sessions (mean number of sessions attended = 25.7, SD = 12.3; b = 2.55, 95% CI: 1.43, 3.68). Other significant predictors were sex and pathway, with females attending fewer sessions than males (b = -1.11, 95% CI: -1.66, -0.55), and those on a level 4 pathway attending fewer sessions than those on the generic pathway (b = -1.51, 95% CI -2.29, -0.75).

#### Adherence: attendance at 16-week consultation

Supplementary Material 6 (Supplementary Table 4) presents the results of the multi-level binary logistic modelling analysis. Only those participants who attended the first exercise session were included in this analysis (*n* = 13,008). Of these, 8,235 (63.3%) attended the 16-week consultation. The breakdown by programme of delivery was as follows: standard programme 5,047 (60.9%), hybrid 2,680 (68.7%) and modified programme 508 (62.4%).

There was no effect of programme of delivery, with the likelihood of attending the 16-week consultation equivalent for patients on all versions of programme. Older patients were more likely to attend the 16-week consultation (OR 1.018, 95% CI: 1.014, 1.022), and an upwards trend was again observed for WIMD quintile. In comparison to those on the generic pathway, those on the back care (OR 0.76, 95% CI: 0.58, 0.99), mental health (OR 0.73, 95% CI: 0.57, 0.93), and weight management (OR 0.72, 95% CI: 0.59, 0.89) pathways were less likely to reach the 16-week programme endpoint. See Supplementary Material 9 for graphs displaying the probability (logit) of attending the 16-week consultation by WIMD quintile and pathway.

#### Cost analysis

Table 4 below presents a summary of the staff time and corresponding costs (pound sterling, cost year 2020–2021 and costing perspective that of NERS budget) of providing NERS via face-to-face delivery (in context of the standard and modified programmes) and via virtual delivery (in context of the hybrid and modified programmes, and the future hypothetical programme). For further detail on how these costings were derived, see Supplementary Material 4. Staff time required for delivery (virtual or face-to-face) under the hybrid and modified programmes was higher due to the increased need to clean equipment in the context of Covid-19. Staff time was also higher for virtual delivery under the modified programme as two ERPs were required to deliver each exercise session (a new requirement for this programme type driven by safety concerns – one ERP leads the session and the other monitors participants for safety). The costs of virtual delivery (hybrid and modified programmes) were not offset by service user fees: face-to-face attendees were charged £2 each, but virtual “attendees” were not charged. The hypothetical programme includes a £2 per person charge as this is likely required in any future model to ensure financial sustainability. Costs per service user for virtual delivery are elevated in the hybrid and modified programmes because of the recorded reductions in class size/attendance resulting in fewer people to ‘split’ the cost across.

**Table 4:** Summary of the time and associated costs (pound sterling, cost year 2020–2021) by mode of delivery. Timings are multiplied (‘2 × ’) where two members of staff support delivery of this sub-activity. Reported values are based on unrounded figures

Programme type	Standard	Hybrid		Modified		Hypothetical
Mode of delivery costed per programme of delivery	Face-to-face	Virtual		Face-to-face	Virtual	Virtual
**Costing summary**		**Live stream/ pre-recorded**	**Check-in meeting**			
Sub-activity delivered by ERP	**Time (mins)**	**Time (mins)**	**Time (mins)**	**Time (mins)**	**Time (mins)**	**Time** **(mins)**
Set up room & equipment	10	5	-	10	5	5
Cleaning equipment – pre-exercise	5	10	-	15	10	5
Exercise session	50	50	-	50	2 × 45	2 × 45
Cleaning equipment – post-exercise	5	10	-	15	10	5
Tidy up room & equipment	10	5	-	10	5	5
Recording and entering attendance	10	5	-	10	10	10
Calling to "Check-in"	-	-	15	-	-	-
**Exercise session costs—all sub-activities delivered by ERP**						
Total time per session	90	85	15	110	130	120
Total cost per session (total time multiplied by hourly rate^a^)	£26.95	£25.46	£4.49	£32.94	£38.93	£35.94
Typical class size^b^/views^c^	9.9	50.3	1	4.0	5.3	5.3
Average attendance income (£2 × average attendance; relevant only for face-to-face and hypothetical scenario)	£19.76	-	-	£8.07	-	£10.66
Cost per session Calculated as: (total cost per session-attendance income)/typical class size	£0.73	£0.51	£4.49	£6.16	£7.31	£4.74
** Service user cost across course** **(32 sessions/ 16 check-ins)**	£23.28	£16.20	£71.87	£197.12	£233.77	£151.79
**Consultation sessions costs**^**d**^						
Total cost per service user, week 0	£26.56	-	-	£30.46	£30.46	£30.46
Total cost per service user, week 16	£15.57	£19.47	-	£17.97	£19.47	£19.47
Total cost per service user, week 52	£11.08	£14.97	-	£13.48	£11.98	£11.98
**Cost per service user for 16-week programme**						
Week 0 consultation; ERP sessions (32); week 32 consultation; check-ins during virtual programme	£65.42	£107.54	-	£245.54	£283.69	£201.71

^a^See Supplementary Material 4 for hourly rate derivation. Derived hourly rate for ERPs: £17.97

^b^Class sizes derived from internal audit data – see Supplementary Material 4 for details

^c^Based on service information about accesses of virtual material – see Supplementary Material 4 for details

^d^Consultation costs here assume ERPs complete associated data entry – see Supplementary Material 4 for details of how these figures were calculated

## Discussion

Programme of delivery had no influence on uptake, with those on the standard programme (fully face-to-face delivery) and those on the modified programme (either face-to-face and/or virtual delivery as determined by Covid-19 risk and personal preference) equally likely to attend both the first consultation and the first exercise session. Programme of delivery did however effect adherence, as measured by the number of exercise sessions attended. Compared to those on the standard programme on average those on the hybrid programme (where face-to-face delivery was replaced by remote delivery in response to Covid-19) attended fewer exercise sessions, whereas those on the modified programme attended more exercise sessions. Sociodemographic factors influenced both uptake and adherence. Being older and living in an area of lower deprivation increased the likelihood of attending the first consultation and of reaching the scheme endpoint. Being female increased the likelihood of programme initiation but was associated with lower exercise attendance. Compared to those referred on to the scheme for prevention purposes, those referred from secondary care were more likely to attend the first consultation but less likely to attend exercise sessions. Those referred for weight management purposes were less likely to take up the scheme. Delivery mode was found to have considerable impact on scheme cost. A typical 16-week cycle of the scheme, delivered outside of a pandemic context, was estimated to cost £65.42 per person when provided face-to-face, but £201.71 per person when provided virtually.

This study makes an important contribution to a growing body of work concerning uptake and adherence to ERSs. Observational studies such as this are vital for assessing the success of real-world delivery of ERSs outside of the additional resources and strict controls of randomised controlled trials (RCTs). In addition to providing a comprehensive assessment of the independent effect of socio-demographic factors on uptake and adherence for one of the largest operating ERSs, this study is the first to examine the impact of different programmes of delivery on these key performance indicators and programme costs. This research is timely in a context of increasing demand on limited public health resources and services, reaffirming the importance of striving to make efficiencies while also ensuring that services are appropriately and equitably used.

In this study, scheme uptake, measured as attendance at the first consultation, was 41% for patients referred onto the standard programme. This is lower than the pooled uptake rate of 66% for ERSs, as reported by a systematic review of observational studies [10], and lower than the 70% uptake previously reported for the same scheme for the year 2017 [25]. This decline in uptake on 2017 figures may reflect differences in the absolute number of referrals. The volume of referrals to this ERS has been increasing steadily over the past decade placing increasing demands on the service. Adherence to the standard version of this ERS, defined in terms of attending a minimum of 75% of the programme, was 51%. This is more in line with existing evidence, with the systematic review by Pavey and colleagues of observational studies reporting a pooled adherence of 49% [10]. The concentration of costs towards the beginning of ERSs, due to the administrative activity of referral and registration, means that drop-out in the early stages of the scheme could have a marked effect on overall cost-effectiveness. Further, poor adherence is likely to limit the potential improvement in health outcomes that could be achieved [21].

The primary aim of this study was to examine the effect of programme of delivery on scheme uptake and adherence. For analyses concerning uptake, the effects of both the standard and modified programmes were compared. Patients on the two programmes were equally likely to take up the scheme, as measured by attendance at the first consultation and the first exercise session. Given that the mode(s) of delivery in operation via each programme will have been discussed between the patient and their ERP at the first consultation, and that this should also have occurred between referrer and patient at referral (as directed by the scheme operators), this is a promising finding. It suggests that changing the format of delivery had no effect on scheme drop-out from the point at which referral was made to the initiation of exercise. What is unknown is whether discussions at the point of referral about the nature of delivery had any effect on referral itself. It may be that the offer of the modified programme, which both simultaneously placed restrictions on how some patients could engage with the programme (if deemed very high risk, only virtual delivery available) whilst providing others with increased flexibility (all patients were allowed to choose virtual delivery if preferred, where available), may have positively and/or negatively influenced decisions about whether to accept referral. Along with collecting data on attendance at the first consultation and the first exercise session, the scheme could consider collecting data on patient acceptance of the scheme at referral. This would provide a more complete picture of scheme uptake and allow hypotheses concerning the effect of programme (and other) factors to be tested.

In comparison to patients on the standard programme, those on the modified programme attended more sessions. When new referrals to this ERS were suspended in response to the unfolding pandemic, patients who had been on the standard programme for less than four weeks, or who had declined the hybrid offer, were given ‘postponed’ status, and re-invited when the modified programme was launched. The higher levels of adherence for the modified group may therefore reflect a core of motivated individuals who were eager to continue the programme following an enforced 12-month break. An alternative explanation is that, in line with self-determination theory [46], providing patients with a choice of delivery mode had the effect of enhancing intrinsic motivation to exercise and in turn levels of engagement. It is not possible to untangle the effect of the change in delivery mode observed in this study from the pandemic context; both explanations are plausible and future research will be necessary to see whether this effect can be replicated in a ‘normal’ context.

Analysis also showed that adherence was lower for patients on the hybrid programme than on the standard programme. The cause of the lower levels of adherence for the hybrid group is similarly difficult to untangle. Whilst it might be expected that waiving the usual £2 session fee would encourage attendance, it is impossible to separate out any positive effects that this might have had from other factors, not least the Covid-19 context. The pandemic not only placed restrictions on what people were permitted to do but also reduced people’s motivation to exercise and their perceptions of capability to be active [47], resulting in significant drops in levels of PA at a national level [9]. It is likely therefore that adherence for some patients within the hybrid cohort was low, not because of the change in delivery mode, but due to experiencing low motivation to exercise. It should however be considered that the mode of delivery itself may have had a negative impact. Those offered the hybrid programme were given the choice of a pre-written script to follow at home or virtual delivery, the latter being either in a pre-recorded or live format. Unfortunately, data were not collected on which format individuals opted for, and so further group comparisons could not be made. It is possible that the lower adherence rate for the hybrid programme results from poor adherence among the subgroup using the pre-written scripts and/or pre-recorded sessions. Systematic review evidence indicates that adherence is higher when exercise programmes are supervised rather than unsupervised [22, 48–51]. Given that support from exercise instructors is known to be associated with ERS attendance [39], this may be a contributing factor. A further factor known to be associated with ERS attendance is exercising with others [39]. While exercising in a live virtual session does enable contact with peers, social interactions may have been reduced or diluted in nature, leading to less positive experiences. Digital exclusion may also have had a negative impact on adherence. Patients opting for virtual delivery may have had a poor experience of sessions because of using unsuitable or outdated devices, having an unstable internet connection, or because of a lack of skills or confidence in using the required platforms. The paradox here is that those individuals most likely to benefit from remote delivery, due to for example caring responsibilities, disability, transport costs, are also those most likely to experience digital exclusion [52, 53]. Once again, further research is required to examine the veracity of this finding outside of the pandemic context.

Levels of uptake and adherence do not only have an impact on the overall effectiveness and cost-effectiveness of ERSs; also of concern is the potential for loss of patients from an ERS to be patterned, that is, for uptake and adherence to be lower for individuals from specific socio-demographic groups, thus introducing and/or widening health inequalities. Analysis undertaken in this study examined the independent effect of several socio-demographic factors on two measures of uptake and adherence commonly reported on in the ERS literature. Four clear patterns are evident which add to existing evidence in this area.

First, this study adds to a consistent body of evidence that women are more likely to take up ERSs than men [21, 25–28]. This is a promising finding both for this programme and for ERSs more broadly, given that patterns in levels of PA in Wales and other Western nations show that women are less likely to be active than men [2, 42]. This finding may be a product of women being greater consumers of health care services than men [54], rather than any active targeting on behalf of referrers, but nonetheless, this is a position that should be capitalised on. While uptake was relatively high, women were however found to be less likely to attend exercise sessions than men. This raises the concern that women may experience more barriers to attendance than men and consequently not to achieve the same benefit from the scheme.

Second, this study adds to the breadth of existing evidence to support a positive association between increasing age and both ERS uptake [21, 26, 28, 33, 34, 39] and adherence [21, 28, 32, 35]. While a small number of studies have found no association between age and uptake [27, 36, 37], given the collective evidence to date, this pattern of findings can be asserted with reasonable confidence. Once again, these are encouraging findings, suggesting that ERSs can be successful in reaching and retaining older people who are typically some of the least active members of society [9].

The third clear pattern concerns the effect of deprivation. In line with other studies examining referral to this ERS, there was no discernible difference in referral for patients across the deprivation quintiles (see Table 1) [21, 25]. While on the face of it this is encouraging, given that individuals living in areas of higher deprivation are known to be higher consumers of healthcare services [55], higher proportions of referrals from these groups to NERS would be expected thus indicating that inequalities are being introduced at referral. This study found that the likelihood of patients attending a first consultation increased in line with a lowering magnitude of deprivation (represented by increasing WIMD quintile). Deprivation was also found to have the same relationship with one of this study’s two measures of adherence, attendance at the 16-week consultation, where the likelihood of attendance was greater for all WIMD quintiles in comparison to quintile one, which represents people living in the most deprived areas of Wales. This adds to a growing body of evidence that deprivation has a negative effect on ERS uptake [21, 25, 28, 33] and adherence [21, 28]. The consistent replication of this finding across different ERSs provides a strong indication that ERSs can serve to widen health inequalities. Taken together, these findings suggest that this scheme is amplifying inequalities. Given that deprivation is associated with lower rates of physical activity and higher rates of chronic illness [56], there is a pressing need to implement strategies to increase the referral of patients from areas of higher deprivation and then to actively support these individuals to enrol on and engage with the scheme. Future research should seek to determine whether efforts to increase uptake and adherence of the scheme by individuals living in areas of higher deprivation would improve its cost-effectiveness. In particular, it may be advantageous to concentrate existing resources on fewer patients who have more to gain from participation.

The final pattern observed concerns referral pathway. For this ERS, pathway provides an indication of the health status of the individual and their motivation for entering the scheme. In the wider literature, similar attempts have been made to examine the influence of patients’ health conditions or status on uptake and adherence but there is a high degree of inconsistency in the findings, likely due to differences in conceptualisation and measurement of this predictor and in the choice of comparison group. In this study, the most prominent pattern of findings was for the patients on the level 4 pathway (those referred from secondary care). While these patients were more likely to take up the scheme in comparison to those on the generic pathway (referred from primary care for prevention purposes), they were however less likely to adhere. This perhaps reflects a level of enhanced motivation among this group to regain health and functional status following treatment in secondary care but then greater barriers to attendance due to for example, pain, mobility issues, health-related anxiety, or a relapse or acute episode of their condition. Underlining the point concerning variations in measurement and analysis, while another study of the same ERS programme also found that uptake was higher for level 4 patients, here the reference group was patients with CHD, and further, only those referred on to the generic pathway were included in the sample (health condition was instead coded using ‘reason for referral’ data rather than ‘pathway’) [25]. There is a clear need for researchers and ERS providers to work together to agree on meaningful comparisons in this area and to ensure that suitable and accurate data are collected for this purpose.

Stakeholders typically implement digital delivery of health interventions with the expectation that they will reduce costs. For example, digital delivery may be expected to support delivery to larger numbers of recipients and to result in reduced overheads (e.g. lower or no room hire costs), leading to lower session costs and costs per service user. Virtual delivery of this scheme raised safety concerns, leading to class sizes being restricted to eight service users and sessions being led by two ERPs (one to lead the class and one to monitor participants for safety). This meant that potential digital savings could not be realised; in fact, virtual delivery was more expensive. In this scheme, virtual delivery did not impact overheads such as room hire; this is however more context dependent, as the NERS budget does not typically bear such costs (they are funded by the hosting local authorities). The choice to charge face-to-face attendees only has obvious budget implications. Charging an equivalent £2 session fee for virtual classes would help to reduce the overall cost per service user (though collecting these ‘virtual’ fees may pose logistical challenges and other costs, in terms of ERP time and infrastructure to support their collection), albeit the total would still be higher than that for face-to-face delivery. At the time of analysis, cost to NERS per service user was higher for the hybrid and modified programmes due to smaller class sizes – if these sizes do not increase, it may impact on the sustainability of the service (particularly with the reduction in income from service user fee). Ensuring attendance at virtual classes meets or nears maximum capacity levels at every session is clearly important if virtual delivery is to continue. With face-to-face classes, the number of potential attendees is limited to the number of service users living locally on the relevant pathway. There are no such limits for virtual delivery however, and programme organisers should therefore consider making virtual classes available to all service users at a national level (as opposed to locally only) to maximise attendance and to help offset costs. Given that evidence concerning the cost-effectiveness of ERSs is inconclusive [20], scheme organisers need to carefully consider whether the increased costs of virtual delivery are acceptable or justifiable. Crucially, this needs to be balanced with the potential for virtual delivery to reduce inequalities in uptake and outcomes by making it more accessible to those who are currently underserved by face-to-face delivery.

### Strengths and limitations

A strength of this work concerns the precision with which effects have been estimated. The large sample means that random variation is minimised. This precision is demonstrated in the narrow confidence intervals observed for odds ratios, although more caution should be adopted where analysis includes groups that are less well represented in the sample, such as those experiencing the modified programme. Of importance however, the large sample size also means that the statistical power of analyses to detect significant effects is high. This means that caution must be adopted when interpreting findings with small effect sizes. Consideration should be given as to whether these findings have practical importance in the real world. The observational nature of this study affords both an inherent strength and limitation to the work. This design means that the study has good external validity, providing a snapshot of real-world ERS delivery that can be used by scheme organisers to inform future delivery. Of import in this study, it should be acknowledged that factors relating to the broader context of the Covid-19 pandemic may have been responsible for the differences observed. The only way to have isolated the effects of programme type on uptake and adherence at the time of the pandemic would have been to have run a randomised controlled trial (RCT). This was not a feasible proposition at a time when the scheme was rapidly adapting to changes to continue supporting patients. This observational study was therefore a pragmatic alternative, capitalising on existing data to draw tentative conclusions about the effect of programme type. Future research should test the effect of programme of delivery on uptake and engagement experimentally. A further limitation is not having examined interactions between predictor variables. This type of evidence would have provided a more granular understanding of uptake and adherence for this ERS. Finally, analysis in this study used data collected by the scheme provider and there was a substantial amount of missing data and data entry errors. While attempts were made to recover missing data and correct errors, where accuracy could not be verified, some data had to be excluded from the analysis.

## Conclusions

We tentatively conclude that providing service users with choice over ERS mode of delivery may increase adherence but that enforcing remote delivery may act to reduce this. Further research outside of a pandemic context is required to test this assertion. The socio-demographic patterning of uptake and adherence observed in this study and others, underlines the importance of focusing finite ERS resources on targeting those most likely to benefit and supporting them to take up and complete the programme. Collectively there is now good evidence to support the assertion that being older and coming from an area of lower deprivation increases the likelihood of both ERS uptake and adherence. There is also good evidence that being female increases the likelihood of ERS uptake and growing evidence that it reduces the likelihood of adherence. Findings from the cost analysis challenge the expectation that digital health interventions are cost saving; for this ERS, virtual delivery substantially increased costs. Continued implementation of this mode of delivery could nonetheless be advantageous if it facilitates uptake and adherence among groups known to be most in need of PA intervention. Ensuring virtual delivery addresses health inequalities rather than exacerbating them will however require targeted effort, planning and monitoring.

### Supplementary Information

Supplementary Material 1

Supplementary Material 2

Supplementary Material 3

Supplementary Material 4

Supplementary Material 5

Supplementary Material 6

Supplementary Material 7

Supplementary Material 8

Supplementary Material 9

## Data Availability

The data that support the findings of this study are owned by Public Health Wales (Health Improvement Division) and restrictions apply to the availability of these data, which were used under license for the current study, and so are not publicly available. Data are however available from the authors upon reasonable request and with permission of Public Health Wales. Point of contact for the data is Dr Katie Newby; email: k.newby@herts.ac.uk. Readers who wish to see the original SPSS output for all analyses presented in this paper are referred to 10.18745/ds.28071.

## Abbreviations

BMI: Body Mass Index
CHD: Coronary Heart Disease
CI: Confidence Interval
ECDA: Ethics Committee with Delegated Authority
ERS: Exercise Referral Scheme
ERP: Exercise Referral Professional
GP: General Practitioner
NHS: National Health Service
OR: Odds Ratio
PA: Physical Activity
PHW: Public Health Wales
PHIRST: Public Health Interventions Responsive Studies Team
QALY: Quality-Adjusted Life-Year
NERS: National Exercise Referral Scheme
NIHR: National Institute for Health and Care Research
RCT: Randomised Controlled Trial
UK: United Kingdom
US: United States
WIMD: Welsh Index of Multiple Deprivation
