# Supplementary material for: Moving an exercise referral scheme to remote delivery during the Covid-19 pandemic: an observational study examining the impact on uptake, adherence, and costs
Source: BMC Public Health. 2024 Aug 27;24:2324. doi: 10.1186/s12889-024-19392-y (PMC11348648; doi:10.1186/s12889-024-19392-y)
Supplement: Supplementary file 1 — Supplementary Material 1 [file 12889_2024_19392_MOESM1_ESM.docx]

Additional file 1. NERS Pathways

Supplementary Table 1. Pathways available within the Welsh National Exercise Referral Scheme (NERS)

| **Pathway** | **Grouping** |
| --- | --- |
| Back care | Back care |
| Cancer | Level 4 |
| Cardiac | Level 4 |
| Falls prevention | Level 4 |
| Generic | Generic |
| Lifestyle (for patients awaiting hip/knee replacement) | Level 4 |
| Mental health | Mental health |
| Pregnancy | Level 4 |
| Pulmonary | Level 4 |
| Stroke | Level 4 |
| Weight management | Weight management |
