# Supplementary material for: Moving an exercise referral scheme to remote delivery during the Covid-19 pandemic: an observational study examining the impact on uptake, adherence, and costs
Source: BMC Public Health. 2024 Aug 27;24:2324. doi: 10.1186/s12889-024-19392-y (PMC11348648; doi:10.1186/s12889-024-19392-y)
Supplement: Supplementary file 3 — Supplementary Material 3 [file 12889_2024_19392_MOESM3_ESM.docx]

# Additional File 3. Data screening flowchart
