# Supplementary material for: Moving an exercise referral scheme to remote delivery during the Covid-19 pandemic: an observational study examining the impact on uptake, adherence, and costs
Source: BMC Public Health. 2024 Aug 27;24:2324. doi: 10.1186/s12889-024-19392-y (PMC11348648; doi:10.1186/s12889-024-19392-y)
Supplement: Supplementary file 4 — Supplementary Material 4 [file 12889_2024_19392_MOESM4_ESM.docx]

Additional File 4. Supplementary detail on costing

- Hourly costs of employment, including overheads of pension and NI contributions (no further overheads were included), were derived separately for each job role – details given in the ‘calculation of hourly rates’ section below. The costing year was 2020-2021 (pound sterling) and the primary costing perspective was that of NERS.
- Exercise sessions were regarded as group activities (or treated as a group activity, where they were pre-recorded). To derive a cost-per-service-user, the cost of delivering a session was divided by the mean class size (or viewing figures for pre-recorded sessions) based on information collected by NERS.
- For face-to-face exercise sessions, service users were asked for a payment of £2 each, but no payment was requested for virtual classes (but such a payment is proposed in the ‘Hypothetical Virtual’ offering in Table 4 of the main paper).
- NERS teams were found to vary by structure (for example, whether they included administrative support) and to adapt their offerings (e.g. focusing on particular types of exercise) for local needs: some of this variation (e.g. data entry by different types of staff) is captured in the supplementary materials below, but otherwise the focus is on a ‘typical’ session.
- The costings reported here are likely under costings – costs which were not considered/included are as follows:
  - Resources and costs that go into developing ‘new’ sessions. It is important to note however, that the general approach to implementing new sessions is a rolling update, rather than whole new timetable offerings etc – so the impact in a ‘typical’ week is likely small. The exception is for the hybrid programme where this ‘development’ cost would have been particularly notable given the sudden significant shift in delivery (i.e. the requirement to deliver *all* sessions virtually).
  - Only staff time costs have been considered. The cost of equipment, venue hire or travel for example has not been included. It is worth noting however that the NERS budget *generally* does *not* pay for venue hire – this is provided by the local authorities from alternative budgets. Further, travel time and costs are minimal – generally ERPs deliver their programmes regularly at designated centres (however, some offerings are delivered closer to service users as part of out-reach and/or supporting those in more rural settings).
- The activities reported here do not capture all activities of NERS and its staff – for example, the cost of activities (other than those already noted above) performed by regional managers and administrative support have not been included. Further, as already mentioned, only ERP costs directly related to delivering exercise sessions and consultations are included; examples of additional activities not included are staff meetings and administration.

**Calculation of hourly rates**

Table 1 below gives average salary figures for 2018 based on data provided by the NERS programme managers in email (dated 12.03.2021). These were inflated to 2020/2021 levels using the ‘Personal Social Services annual percentage increases’ taken from Section 15.4.2 of Jones and Burns (2021) (specifically, annual percentage increase on previous year, so, 3.2% and 1.3% prospectively – giving an inflation multiplier of 1.032×1.013=1.051494).

Subsequently, to calculate an hourly rate, we have assumed 225 working days (45 weeks) in a year (figure used in Jones and Burns (2021)) and full time hours of 35 hours per week: this gives 1,575 hour worked per year. Thus, annual salaries were divided by 1,575 to give the hourly rates reported in Table 4 of the main paper.

**Calculation of average face-to-face class size**

Average face-to-face class size for the standard programme was based on audit data collected during October 2019 (7^th^-13^th^), provided by the NERS programme managers in email (dated 15.03.2022). This data is given in Table 2 below. This data consisted of, for each region, the number of classes delivered and the mean class size; from these, total numbers of attendees were calculated (classes delivered multiplied by mean class size) for each region. We summed these figures across all regions to give 11,265 attendees across Wales in 1,140 classes during this period, resulting in a mean attendance of 9.9 people per class (11,265÷1,140).

Average face-to-face class size for the modified programme was based on service data sourced from NERS programme managers. This is shown in Table 4 below and consisted of the monthly numbers of face-to-face sessions and attendances for July 2021 to January 2022. For this period, we calculated a total of 51,856 attendances across Wales in 12,845 face-to-face sessions; this gives a mean class size of 4.0 (51,856÷12,845).

**Calculation of average virtual views/class size**

The mean number of views/attendees for the virtual classes under the hybrid programme is based on service data sourced from the NERS programme managers. This data is presented in Table 3 below, for period October 2020 to June 2021, giving monthly numbers of exercise videos recorded, live virtual classes delivered and the number of views of the exercise sessions. Consequently, for this period in Wales, we calculate a total of 374,529 views of 7,447 sessions, giving a mean 50.3 (374,529÷7,447) views of each exercise session.

The mean class size for virtual delivery under the modified programme is based on service data sourced from the programme managers. This data is presented in Table 5 below: for the period July 2021 to January 2022, monthly totals of virtual sessions and the number of live accesses. From this, we calculate that across Wales for the period there were 11,266 access of 2,114 virtual sessions, giving a mean class size of 5.3 (11,266÷2,114) for virtual sessions under the modified programme. The same class size was assumed for the hypothetical programme.

**Further costing detail**

Table 4 presented in the main paper is a summary of the costing work. Further detail for costing ERP sessions is given in Table 6 below, including variations for different types of the NERS team – for example, where data entry is conducted by administrative staff. Staff time and associated costs of delivering consultation sessions at weeks 0, 16 and 52 are given in Tables 7, 9 and 10 below respectively. A group-based approach to consultation at week 0 under the standard programme is given in Table 8 below – this approach is no longer utilised.

**Supplementary Table 1**: Salary and hourly rates used in the NERS costing

| **Position** | **2018/2019 values** | | **2020/2021 values** | |
| --- | --- | --- | --- | --- |
|  | **Mid-range salary** | **Mid-range including overheads** | **Mid-range including overheads** | **Hourly rate** |
| Coordinator | £28,609 | £36,073 | £37,930 | £24.08 |
| ERP | £21,393 | £26,914 | £28,300 | £17.97 |
| Admin support | £18,533 | £23,198 | £24,393 | £15.49 |

**Supplementary Table 2**: Audit data for standard programme giving number of face-to-face classes delivered, number of attendees and mean class size for October 7^th^ – 13^th^ 2019

| **Region** | **Classes delivered** | **Attendees*** | **Mean class size** |
| --- | --- | --- | --- |
| Bridgend | 77 | 460 | 6.0 |
| Neath Port Talbot | 70 | 703 | 10.0 |
| Swansea | 50 | 364 | 7.3 |
| Merthyr Tydfil | 33 | 244 | 7.4 |
| Rhondda Cynon Taf | 48 | 475 | 9.9 |
| Powys | 34 | 218 | 6.4 |
| Anglesey | 46 | 239 | 5.2 |
| Conwy | 48 | 274 | 5.7 |
| Denbigshire | 53 | 377 | 7.1 |
| Flintshire | 35 | 350 | 10.0 |
| Gwynedd | 41 | 293 | 7.1 |
| Wrexham | 50 | 404 | 8.1 |
| Cardiff | 36 | 803 | 22.3 |
| Vale of Glamorgan | 48 | 379 | 7.9 |
| Carmarthen | 56 | 323 | 5.8 |
| Ceredigion | 56 | 746 | 13.3 |
| Pembrokeshire | 56 | 620 | 11.1 |
| Blaenau Gwent | 28 | 632 | 22.6 |
| Caerphilly | 51 | 936 | 18.4 |
| Monmouthshire | 100 | 1,100 | 11.0 |
| Newport | 39 | 825 | 21.2 |
| Torfaen | 85 | 502 | 5.9 |
| **Total** | **1,140** | **11,265** | **9.9** |

* Attendees estimated from classes delivered multiplied by mean class size and rounded to the nearest integer

**Supplementary Table 3**: Data for hybrid programme giving accesses of virtual exercise material, either live or pre-recorded

| **Month** | **Exercise videos recorded** | **Live virtual classes delivered** | **Total exercise sessions delivered** | **Views of exercise sessions** | **Mean views per virtual offering** |
| --- | --- | --- | --- | --- | --- |
| Oct-20 | 202 | 459 | 661 | 71,392 | 108.0 |
| Nov-20 | 207 | 590 | 797 | 60,150 | 75.5 |
| Dec-20 | 153 | 445 | 598 | 44,515 | 74.4 |
| Jan-21 | 167 | 655 | 822 | 39,861 | 48.5 |
| Feb-21 | 186 | 695 | 881 | 32,509 | 36.9 |
| Mar-21 | 227 | 828 | 1,055 | 39,967 | 37.9 |
| Apr-21 | 192 | 711 | 903 | 33,834 | 37.5 |
| May-21 | 199 | 698 | 897 | 26,636 | 29.7 |
| Jun-21 | 141 | 692 | 833 | 25,665 | 30.8 |
| **Total** | 1,674 | 5,773 | 7,447 | 374,529 | **50.3** |

**Supplementary Table 4**: Data for the modified programme giving face-to-face sessions (indoor and outdoor combined)

| **Month** | **Number of face-to-face sessions delivered** | **Numbers of attendances** | **Mean class size** |
| --- | --- | --- | --- |
| Jul-21 | 1,395 | 4,202 | 3.0 |
| Aug-21 | 1,913 | 6,620 | 3.5 |
| Sep-21 | 2,088 | 8,835 | 4.2 |
| Oct-21 | 2,176 | 9,915 | 4.6 |
| Nov-21 | 1,892 | 7,324 | 3.9 |
| Dec-21 | 1,395 | 5,715 | 4.1 |
| Jan-22 | 1,986 | 9,245 | 4.7 |
| **Total** | 12,845 | 51,856 | **4.0** |

**Supplementary Table 5**: Data for the modified programme giving views of live-streamed material

| **Month** | **Virtual sessions delivered live** | **Total live accesses** | **Mean class size** |
| --- | --- | --- | --- |
| Jul-21 | 520 | 2,669 | 5.1 |
| Aug-21 | 347 | 1,651 | 4.8 |
| Sep-21 | 339 | 1,581 | 4.7 |
| Oct-21 | 240 | 1,515 | 6.3 |
| Nov-21 | 227 | 897 | 4.0 |
| Dec-21 | 160 | 809 | 5.1 |
| Jan-22 | 281 | 2,144 | 7.6 |
| **Total** | 2,114 | 11,266 | **5.3** |

**Supplementary Table 6**: Detailed costings for exercise sessions, with different costings depending on staff delivering sub-activity

| **Programme type** | | **Standard** | | **Hybrid** | | | | **Modified** | | | | |
| --- | --- | --- | --- | --- | --- | --- | --- | --- | --- | --- | --- | --- |
| Mode of delivery costed | | Face-to-face | | Virtual | | | | Face-to-face | | Virtual | | |
| **ERP detailed costing** | |  | | **Live stream/ pre-recorded** | | **Check-in/IT support** | |  | | |  | |
| Sub-activity | Delivery options | **Time (mins)** | **Range (mins)** | **Time (mins)** | **Range** | **Time (mins)** | **Range (mins)** | **Time (mins)** | **Range (mins)** | | **Time (mins)** | **Range** |
| Set up room & equipment | ERP | 10 | 0-15 | 5 | 0 |  |  | 10 | 0-15 | | 5 |  |
|  | Other | 10 | 0-15 |  |  |  |  | 10 | 0-15 | |  |  |
| Cleaning equipment | ERP | 5 | 5-10 | 10 | 5-10 |  |  | 15 | 5-15 | | 10 | 5-10 |
|  | Other | 5 | 5-10 |  |  |  |  | 15 | 5-15 | |  |  |
| Exercise session | ERP | 50 | 45-60 | 50 | 45-60 |  |  | 50 | 45-60 | | 90 | 45-60 |
| Post cleaning equipment | ERP | 5 | 5-10 | 10 | 5-10 |  |  | 15 | 5-15 | | 10 | 5-10 |
|  | Other | 5 | 5-10 |  |  |  |  | 15 | 5-15 | |  |  |
| Tidy up room & equipment | ERP | 10 | 10-15 | 5 | 10-15 |  |  | 10 | 10-15 | | 5 | 10-15 |
|  | Other | 10 | 10-15 |  |  |  |  | 10 | 10-15 | |  |  |
| Recording and entering attendance | ERP | 10 | 5-10 | 5 | 5-10 |  |  | 10 | 5-10 | | 10 | 5-10 |
| Recording and entering attendance | Admin supp. | 10 | 5-10 | 5 | 5-10 |  |  | 10 | 5-10 | | 10 | 5-10 |
| Calling to support IT or "Check-in" | ERP |  |  |  |  | 15 | 10-15 |  |  | |  |  |
| **ERP session - all sub-activities delivered by ERP** |  |  | |  | |  | |  | | |  | |
| Total time per sess. |  | 90 | | 85 | | 15 | | 110 | | | 130 | |
| Total cost per sess. |  | £26.95 | | £25.46 | | £4.49 | | £32.94 | | | £38.93 | |
| Typical class size/views |  | 9.9 | | 50.3 | | 1 | | 4.0 | | | 5.3 | |
| Attendance income (£2/person/F2F sess.) |  | £19.76 | | £0.00 | | £0.00 | | £8.07 | | | £0.00 | |
| Cost per session |  | £0.73 | | £0.51 | | £4.49 | | £6.16 | | | £7.31 | |
| **Client cost across course (32 sessions/ 16 check-ins)** |  | £23.28 | | £16.20 | | £71.87 | | £197.12 | | | £233.77 | |
| **ERP session - admin data entry** |  |  | |  | |  | |  | | |  | |
| ERP time total per session |  | 80 | | 80 | |  | | 100 | | | 120 | |
| Admin time total per session |  | 10 | | 5 | |  | | 10 | | | 10 | |
| Total time per sess. |  | 90 | | 85 | |  | | 110 | | | 130 | |
| ERP cost per session |  | £23.96 | | £23.96 | |  | | £29.95 | | | £35.94 | |
| Admin cost per sess. |  | £2.58 | | £1.29 | |  | | £2.58 | | | £2.58 | |
| Total cost per sess. |  | £26.54 | | £25.25 | |  | | £32.53 | | | £38.52 | |
| Typical class size/views |  | 9.9 | | 50.3 | |  | | 4.0 | | | 5.3 | |
| Attendance income (£2/person/F2F sess.) |  | £19.76 | | £0.00 | |  | | £8.07 | | | £0.00 | |
| Cost per session |  | £0.69 | | £0.50 | |  | | £6.06 | | | £7.23 | |
| **Client course cost (32 sess./16 check-ins)** |  | £21.94 | | £16.07 | |  | | £193.84 | | | £231.29 | |
| **ERP session - admin data entry & support by leisure centre** |  |  | |  | |  | |  | | |  | |
| ERP time total per session |  | 50 | | 80 | |  | | 50 | | | 120 | |
| Support by leisure centre staff |  | 30 | | 0 | |  | | 50 | | | 0 | |
| Admin time total per session |  | 10 | | 5 | |  | | 10 | | | 10 | |
| Total time per sess. |  | 90 | | 85 | |  | | 110 | | | 130 | |
| ERP cost per session |  | £14.97 | | £23.96 | |  | | £14.97 | | | £35.94 | |
| Leisure centre staff costs |  | £0.00 | | £0.00 | |  | | £0.00 | | | £0.00 | |
| Admin cost per session |  | £2.58 | | £1.29 | |  | | £2.58 | | | £2.58 | |
| Total cost per sess. |  | £17.56 | | £25.25 | |  | | £17.56 | | | £38.52 | |
| Typical class size/views |  | 9.9 | | 50.3 | |  | | 4.0 | | | 5.3 | |
| Attendance income (£2/person/F2F sess.) |  | £19.76 | | £0.00 | |  | | £8.07 | | | £0.00 | |
| Cost per session |  | -£0.22 | | £0.50 | |  | | £2.35 | | | £7.23 | |
| **Client course cost (32 sess./16 check-ins)** |  | -£7.15 | | £16.07 | |  | | £75.15 | | | £231.29 | |

**Supplementary Table 7**: Detailed costings for consultation at week 0

| **Programme type** | | | **Standard** | | **Hybrid** | | **Modified** | | | |
| --- | --- | --- | --- | --- | --- | --- | --- | --- | --- | --- |
| Mode of delivery costed | | | Face-to-face | | Virtual | | Face-to-face | | Virtual | |
| **Consultation at week 0** | | |  | | **Live stream/ pre-recorded** | |  | |  | |
| Sub-activity | | Delivery options | **Time (mins)** | **Range (mins)** | **Time (mins)** | **Range (mins)** | **Time (mins)** | **Range (mins)** | **Time (mins)** | **Range (mins)** |
| Allocating ERP to client | Coord. | | 5 | 5-10 |  |  | 5 | 5-10 | 5 | 5-10 |
| Engaging client (initial contact; arranging appointment) | ERP | | 5 | 5-20 |  |  | 5 | 5-20 | 15 | 5-20 |
| Consultation with client | ERP | | 60 | 30-90 |  |  | 60 | 30-90 | 60 | 30-90 |
| Cleaning the room | ERP | | 2 | 0 |  |  | 15 | 10-15 | 5 | 5-10 |
| Data entry | ERP | | 15 | 15-25 |  |  | 15 | 15-25 | 15 | 15-25 |
|  | Admin | | 15 | 10-20 |  |  | 15 | 10-20 | 15 | 10-20 |
| **1-2-1 consultation - ERP data entry** |  | |  | |  |  |  | |  | |
| Coordinator time total |  | | 5 | |  | | 5 | | 5 | |
| ERP time total |  | | 82 | |  | | 95 | | 95 | |
| Total time per client |  | | 87 | |  | | 100 | | 100 | |
| Coordinator cost |  | | £2.01 | |  | | £2.01 | | £2.01 | |
| ERP cost |  | | £24.56 | |  | | £28.45 | | £28.45 | |
| Total cost per client |  | | £26.56 | |  | | £30.46 | | £30.46 | |
| **1-2-1 consultation - admin data entry** | | |  | |  | |  | |  | |
| Coordinator time total |  | | 5 | |  | | 5 | | 5 | |
| ERP time total |  | | 67 | |  | | 80 | | 80 | |
| Admin support time total |  | | 15 | |  | | 15 | | 15 | |
| Total time per client |  | | 87 | |  | | 100 | | 100 | |
| Coordinator cost |  | | £2.01 | |  | | £2.01 | | £2.01 | |
| ERP cost |  | | £20.06 | |  | | £23.96 | | £23.96 | |
| Admin cost |  | | £3.87 | |  | | £3.87 | | £3.87 | |
| Total cost per client |  | | £25.94 | |  | | £29.84 | | £29.84 | |

**Supplementary Table 8**: Detailed costings for *group* consultation at week 0. Not offered during the remote or modified programme

| **Consultation at week 0: Group** | | **Standard** | |
| --- | --- | --- | --- |
|  |  |  | |
| Sub-activity | Delivery options | **Time (mins)** | **Range (mins)** |
| Coordinator allocating ERP | Coordinator | 20 | 40-80 |
| Engaging service users | ERP | 40 | 40-80 |
| Meeting with service users for consultation | ERP | 90 | 60-120 |
| Cleaning the room | ERP | 10 | 0 |
| Data entry (ERP) | ERP | 120 | 90-180 |
|  | Admin support | 120 | 90-180 |
| **Group consultation - ERP data entry** |  |  | |
| Coordinator time total |  | 20 | |
| ERP time total |  | 260 | |
| Total time |  | 280 | |
| Coordinator cost |  | £8.03 | |
| ERP cost |  | £77.86 | |
| Total cost |  | £85.89 | |
| Total cost per service user |  | £10.74 | |
| **Group consultation - admin data entry** | |  | |
| Coordinator time total |  | 20 | |
| ERP time total |  | 140 | |
| Admin support time total |  | 120 | |
| Total time |  | 280 | |
| Coordinator cost |  | £8.03 | |
| ERP cost |  | £41.93 | |
| Admin cost |  | £30.97 | |
| Total cost |  | £80.93 | |
| Cost per service user |  | £10.12 | |

**Supplementary Table 9**: Detailed costings for consultation at week 16

| **Programme type** | | **Standard** | | **Hybrid** | | **Modified** | | | |
| --- | --- | --- | --- | --- | --- | --- | --- | --- | --- |
| Mode of delivery costed | | Face-to-face | | Virtual | | Face-to-face | | Virtual | |
| **Consultation at week 16** | |  | | **Live stream/ pre-recorded** | |  | |  | |
| Sub-activity | Delivery options | **Time (mins)** | **Range** | **Time (mins)** | **Range** | **Time (mins)** | **Range (mins)** | **Time (mins)** | **Range (mins)** |
| Engaging client (initial contact; arranging appointment) | ERP | 5 | 5-20 | 15 | 15-25 | 5 | 5-20 | 15 | 15-25 |
| Consultation with client | ERP | 30 | 30-45 | 30 | 30-45 | 30 | 30-45 | 30 | 30-45 |
| Room cleaning | ERP | 2 |  | 5 | 5-10 | 10 | 5-10 | 5 | 5-10 |
| Data entry (ERP) | ERP | 15 | 15-25 | 15 | 15-25 | 15 | 15-25 | 15 | 15-25 |
|  | Admin | 15 | 10-20 | 15 | 10-20 | 15 | 10-20 | 15 | 10-20 |
| **1-2-1 consultation - ERP data entry** |  |  | |  | |  | |  | |
| Total time per client |  | 52 | | 65 | | 60 | | 65 | |
| Total cost per client |  | £15.57 | | £19.47 | | £17.97 | | £19.47 | |
| **1-2-1 consultation - admin data entry** | |  | |  | |  | |  | |
| ERP time total |  | 37 | | 50 | | 45 | | 50 | |
| Admin support time total |  | 15 | | 15 | | 15 | | 15 | |
| Total time per client |  | 52 | | 65 | | 60 | | 65 | |
| ERP cost |  | £11.08 | | £14.97 | | £13.48 | | £14.97 | |
| Admin cost |  | £3.87 | | £3.87 | | £3.87 | | £3.87 | |
| Total cost per client |  | £14.95 | | £18.85 | | £17.35 | | £18.85 | |

**Supplementary Table 10:** Detailed costings for consultation at week 52

| **Programme type** | | **Standard** | | **Hybrid** | | **Modified** | | | | |
| --- | --- | --- | --- | --- | --- | --- | --- | --- | --- | --- |
| Mode of delivery costed | | Face-to-face | | Virtual | | Face-to-face | | Virtual | | |
| **Consultation at week 52** | |  | | **Live stream/ pre-recorded** | |  | | |  | |
| Sub-activity | Delivery options | **Time (mins)** | **Range** | **Time (mins)** | **Range** | **Time (mins)** | **Range** | | **Time (mins)** | **Range (mins)** |
| Engaging client (initial contact; arranging appointment) | ERP | 5 | 5-20 | 15 | 15-25 | 5 | 5-20 | | 5 | 2-20 |
| Consultation with client | ERP | 20 | 15-45 | 20 | 15-45 | 20 | 15-45 | | 20 | 15-45 |
| Cleaning the room | ERP | 2 |  | 5 | 5-10 | 10 | 5-10 | | 5 | 5-10 |
| Data entry (ERP) | ERP | 10 | 10-15 | 10 | 10-15 | 10 | 10-15 | | 10 | 10-15 |
|  | Admin | 10 | 10-15 | 10 | 10-15 | 10 | 10-15 | | 10 | 10-15 |
| **1-2-1 consultation - ERP data entry** |  |  | |  | |  | | |  | |
| Total time per client |  | 37 | | 50 | | 45 | | | 40 | |
| Total cost per client |  | £11.08 | | £14.97 | | £13.48 | | | £11.98 | |
| **1-2-1 consultation - admin data entry** | |  | |  | |  | | |  | |
| ERP time total |  | 27 | | 40 | | 35 | | | 30 | |
| Admin support time total |  | 10 | | 10 | | 10 | | | 10 | |
| Total time per client |  | 37 | | 50 | | 45 | | | 40 | |
| ERP cost |  | £8.09 | | £11.98 | | £10.48 | | | £8.98 | |
| Admin cost |  | £2.58 | | £2.58 | | £2.58 | | | £2.58 | |
| Total cost per client |  | £10.67 | | £14.56 | | £13.06 | | | £11.57 | |

**References**

Jones, K, and Amanda Burns. 2021. *Unit Costs of Health & Social Care 2021* (Personal Social Services Research Unit, University of Kent: Coventry).
