# Supplementary material for: Moving an exercise referral scheme to remote delivery during the Covid-19 pandemic: an observational study examining the impact on uptake, adherence, and costs
Source: BMC Public Health. 2024 Aug 27;24:2324. doi: 10.1186/s12889-024-19392-y (PMC11348648; doi:10.1186/s12889-024-19392-y)
Supplement: Supplementary file 5 — Supplementary Material 5 [file 12889_2024_19392_MOESM5_ESM.docx]

Additional File 5. Demographic characteristics of patients referred

Supplementary Table 1. Characteristics of all patients referred to NERS between 2019 and 2021 (n=37,960)

| **Characteristic** |  |  |
| --- | --- | --- |
|  | **Mean** | **Standard deviation** |
| **Age** | 56.5 | 17.5 |
|  | **n** | **%** |
| **Sex** |  |  |
| Female | 24,008 | 63.2 |
| Male | 13,951 | 36.8 |
| Missing | 1 |  |
|  |  |  |
| **WIMD quintile** |  |  |
| 1 (most deprived) | 7,108 | 19.2 |
| 2 | 7,578 | 20.5 |
| 3 | 7,885 | 21.3 |
| 4 | 7,831 | 21.2 |
| 5 (least deprived) | 6,560 | 17.7 |
| Missing | 998 |  |
|  |  |  |
| **Local health board** |  |  |
| Aneurin Bevan | 6,561 | 17.3 |
| Betsi Cadwallader | 11,429 | 30.1 |
| Cardiff and the Vale | 5,172 | 13.6 |
| Cwm Taf | 5,403 | 14.2 |
| Hywel Dda | 4,317 | 11.4 |
| Powys | 1,499 | 3.9 |
| Swansea Bay UHB | 3,577 | 9.4 |
| Missing | 2 |  |
|  |  |  |
| **Referral reason** |  |  |
| Back care | 1,352 | 3.6 |
| Generic | 21,552 | 56.8 |
| Level 4 | 6,495 | 17.1 |
| Mental health | 2,710 | 7.1 |
| Weight management | 5,851 | 15.4 |
| Missing | 0 |  |
|  |  |  |
| **Referrer type** |  |  |
| GP | 15,066 | 39.7 |
| Physiotherapist | 12,219 | 32.2 |
| Practice nurse | 4,544 | 12.0 |
| Other | 6,130 | 16.1 |
| Missing | 1 |  |
