# Supplementary material for: Moving an exercise referral scheme to remote delivery during the Covid-19 pandemic: an observational study examining the impact on uptake, adherence, and costs
Source: BMC Public Health. 2024 Aug 27;24:2324. doi: 10.1186/s12889-024-19392-y (PMC11348648; doi:10.1186/s12889-024-19392-y)
Supplement: Supplementary file 6 — Supplementary Material 6 [file 12889_2024_19392_MOESM6_ESM.docx]

Additional File 6. Results of all multivariate analysis

Supplementary Table 1. Multilevel binary logistic regression results for uptake measured as attendance at first consultation (uptake 1)

| **Characteristic** | **OR** | **Coefficient b** | **p-value** | **95% Confidence interval** | |
| --- | --- | --- | --- | --- | --- |
|  |  |  |  | Lower limit | Upper limit |
|  |  |  |  |  |  |
| **Programme** (baseline Standard) |  |  |  |  |  |
| Modified | 0.95 |  | 0.76 | 0.70 | 1.30 |
|  |  |  |  |  |  |
| **Sex** (baseline Male) |  |  |  |  |  |
| Female | 1.10 |  | **0.03** | 1.01 | 1.20 |
|  |  |  |  |  |  |
| **Age** |  | 0.019 | **<0.001** | 0.01 | 0.02 |
|  |  |  |  |  |  |
| **WIMD quintile** (baseline 1 – most deprived) |  |  |  |  |  |
| 2 | 1.08 |  | 0.09 | 0.99 | 1.19 |
| 3 | 1.15 |  | **<0.01** | 1.05 | 1.27 |
| 4 | 1.17 |  | **0.001** | 1.06 | 1.28 |
| 5 (least deprived) | 1.25 |  | **<0.001** | 1.13 | 1.38 |
|  |  |  |  |  |  |
| **Pathway** (baseline Generic) |  |  |  |  |  |
| Back care | 1.10 |  | 0.44 | 0.70 | 1.38 |
| Level 4 | 1.24 |  | **0.02** | 1.04 | 1.48 |
| Mental health | 0.85 |  | 0.11 | 0.69 | 1.04 |
| Weight management | 0.83 |  | **0.04** | 0.69 | 0.99 |
|  |  |  |  |  |  |
| **Referrer** (baseline GP) |  |  |  |  |  |
| Physiotherapist | 1.03 |  | 0.70 | 0.88 | 1.20 |
| Practice nurse | 1.10 |  | 0.27 | 0.93 | 1.30 |
| Other | 1.03 |  | 0.74 | 0.87 | 1.21 |
|  |  |  |  |  |  |

Bold values signify significant findings (p<0.05)

Supplementary Table 2 Multilevel binary logistic regression results for uptake measured as attendance at first exercise session (uptake 2)

| **Characteristic** | **OR** | **Coefficient b** | **p-value** | **95% Confidence interval** | |
| --- | --- | --- | --- | --- | --- |
|  |  |  |  | Lower limit | Upper limit |
|  |  |  |  |  |  |
| **Programme** (baseline Standard) |  |  |  |  |  |
| Modified | 1.14 |  | 0.64 | 0.66 | 1.96 |
|  |  |  |  |  |  |
| **Sex** (baseline Male) |  |  |  |  |  |
| Female | 1.11 |  | 0.29 | 0.91 | 1.36 |
|  |  |  |  |  |  |
| **Age** |  | 0.012 | **<0.001** | 0.006 | 0.017 |
|  |  |  |  |  |  |
| **WIMD quintile** (baseline 1 – most deprived) |  |  |  |  |  |
| 2 | 1.02 |  | 0.88 | 0.77 | 1.35 |
| 3 | 1.28 |  | 0.10 | 0.96 | 1.70 |
| 4 | 1.16 |  | 0.33 | 0.87 | 1.54 |
| 5 (least deprived) | 1.31 |  | 0.08 | 0.97 | 1.78 |
|  |  |  |  |  |  |
| **Pathway** (baseline Generic) |  |  |  |  |  |
| Back care | 0.96 |  | 0.84 | 0.64 | 1.44 |
| Level 4 | 1.16 |  | 0.27 | 0.89 | 1.50 |
| Mental health | 0.90 |  | 0.53 | 0.64 | 1.26 |
| Weight management | 0.83 |  | 0.19 | 0.64 | 1.09 |
|  |  |  |  |  |  |

Bold values signify significant findings (p<0.05)

Supplementary Table 3 Multilevel linear regression results for engagement measured as the total number of exercise sessions attended over the 16-week programme (adherence 1)

| **Characteristic** | **Coefficient b** | **p-value** | **95% Confidence interval** | |
| --- | --- | --- | --- | --- |
|  |  |  | Lower limit | Upper limit |
|  |  |  |  |  |
| **Programme** (baseline Standard) |  |  |  |  |
| Hybrid | -3.81 | **<0.001** | -4.45 | -3.16 |
| Modified | 2.55 | **<0.001** | 1.43 | 3.68 |
|  |  |  |  |  |
| **Sex** (baseline Male) |  |  |  |  |
| Female | -1.11 | **<0.001** | -1.66 | -0.55 |
|  |  |  |  |  |
| **Age** (per year) | 0.01 | 0.34 | -0.01 | 0.03 |
|  |  |  |  |  |
| **WIMD quintile** (baseline 1 – most deprived) |  |  |  |  |
| 2 | -0.41 | 0.39 | -1.34 | 0.52 |
| 3 | 0.28 | 0.56 | -0.65 | 1.20 |
| 4 | -0.54 | 0.25 | -1.46 | 0.39 |
| 5 (least deprived) | 0.14 | 0.78 | -0.81 | 1.08 |
|  |  |  |  |  |
| **Pathway** (baseline Generic) |  |  |  |  |
| Back care | -1.29 | 0.18 | -0.27 | -2.74 |
| Level 4 | -1.51 | **<0.001** | -2.29 | -0.75 |
| Mental health | -0.71 | 0.26 | -1.95 | 0.54 |
| Weight management | -0.57 | 0.19 | -1.44 | 0.29 |

Bold values signify significant findings (p<0.05)

Supplementary Table 4 Multilevel binary logistic regression results for retention measured as attendance at 16-week consultation (adherence 2)

| **Characteristic** | **OR** | **p-value** | **95% Confidence interval** | |
| --- | --- | --- | --- | --- |
|  |  |  | Lower limit | Upper limit |
|  |  |  |  |  |
| **Programme** (baseline Standard) |  |  |  |  |
| Hybrid | 1.52 | 0.15 | 0.86 | 2.70 |
| Modified | 0.89 | 0.71 | 0.48 | 1.64 |
|  |  |  |  |  |
| **Sex** (baseline Male) | 0.96 | 0.29 | 0.88 | 1.04 |
| Female |  |  |  |  |
|  |  |  |  |  |
| **Age** | 1.02 | **<0.001** | 1.01 | 1.02 |
|  |  |  |  |  |
| **WIMD quintile** (baseline 1 – most deprived) |  |  |  |  |
| 2 | 1.18 | **0.04** | 1.00 | 1.39 |
| 3 | 1.31 | **<0.01** | 1.12 | 1.55 |
| 4 | 1.40 | **<0.001** | 1.19 | 1.65 |
| 5 (least deprived) | 1.39 | **<0.001** | 1.17 | 1.66 |
|  |  |  |  |  |
| **Pathway** (baseline Generic) |  |  |  |  |
| Back care | 0.76 | **0.05** | 0.58 | 0.99 |
| Level 4 | 0.84 | 0.07 | 0.70 | 1.01 |
| Mental health | 0.73 | **0.01** | 0.57 | 0.93 |
| Weight management | 0.72 | **<0.01** | 0.59 | 0.89 |
|  |  |  |  |  |

Bold values signify significant findings (p<0.05)
