# Supplementary material for: Moving an exercise referral scheme to remote delivery during the Covid-19 pandemic: an observational study examining the impact on uptake, adherence, and costs
Source: BMC Public Health. 2024 Aug 27;24:2324. doi: 10.1186/s12889-024-19392-y (PMC11348648; doi:10.1186/s12889-024-19392-y)
Supplement: Supplementary file 7 — Supplementary Material 7 [file 12889_2024_19392_MOESM7_ESM.docx]

Additional File 7. Graphs displaying probability of attending first consultation by selected demographic characteristics

Probability (logit) of attending first consultation predicted by sex, pathway, and WIMD quintile

| 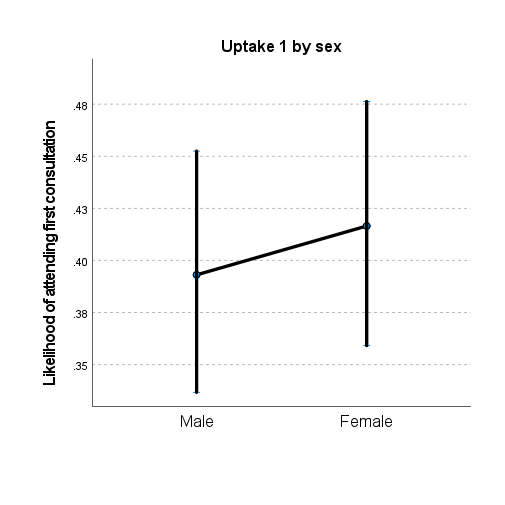 | 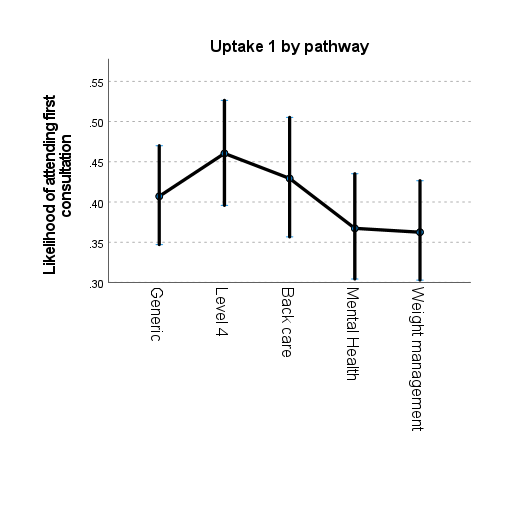 |
| --- | --- |
| 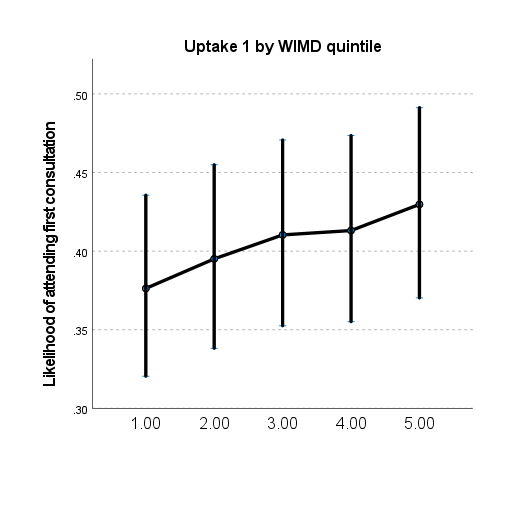 |  |
