# Supplementary material for: Moving an exercise referral scheme to remote delivery during the Covid-19 pandemic: an observational study examining the impact on uptake, adherence, and costs
Source: BMC Public Health. 2024 Aug 27;24:2324. doi: 10.1186/s12889-024-19392-y (PMC11348648; doi:10.1186/s12889-024-19392-y)
Supplement: Supplementary file 8 — Supplementary Material 8 [file 12889_2024_19392_MOESM8_ESM.docx]

Additional File 8. Graph displaying distribution of exercise sessions

## Frequency distribution for the number of recorded sessions attended by patients completing the scheme


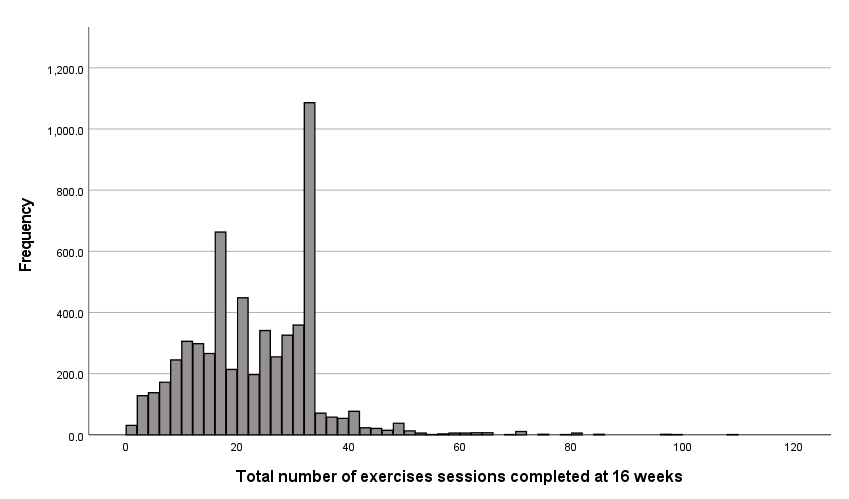


Peaks are observed at 16 weeks and 32 weeks which likely reflect groups of patients choosing to attend once and twice per week respectively over the 16-week programme.
