# Supplementary material for: Moving an exercise referral scheme to remote delivery during the Covid-19 pandemic: an observational study examining the impact on uptake, adherence, and costs
Source: BMC Public Health. 2024 Aug 27;24:2324. doi: 10.1186/s12889-024-19392-y (PMC11348648; doi:10.1186/s12889-024-19392-y)

Additional File 9. Graphs displaying the probability of attending the 16-week consultation by selected demographic characteristics

Probability (logit) of attending the 16-week consultation predicted by pathway and WIMD quintile


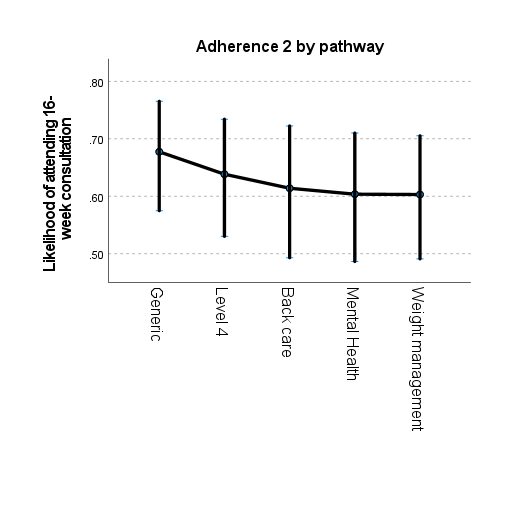


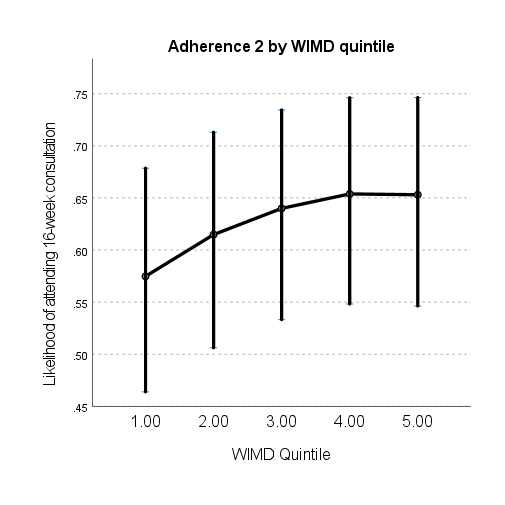

Supplement: Supplementary file 9 — Supplementary Material 9 [file 12889_2024_19392_MOESM9_ESM.docx]
